# The Tsetse Metabolic Gambit: Living on Blood by Relying on Symbionts Demands Synchronization

**DOI:** 10.3389/fmicb.2022.905826

**Published:** 2022-06-09

**Authors:** Mason H. Lee, Miguel Medina Munoz, Rita V. M. Rio

**Affiliations:** ^1^Department of Biology, Eberly College of Arts and Sciences, West Virginia University, Morgantown, WV, United States; ^2^Department of Bacteriology, College of Agricultural and Life Sciences, University of Wisconsin-Madison, Madison, WI, United States

**Keywords:** tsetse, *Wigglesworthia*, microbiota, insect, epigenetics

## Abstract

Tsetse flies have socioeconomic significance as the obligate vector of multiple *Trypanosoma* parasites, the causative agents of Human and Animal African Trypanosomiases. Like many animals subsisting on a limited diet, microbial symbiosis is key to supplementing nutrient deficiencies necessary for metabolic, reproductive, and immune functions. Extensive studies on the microbiota in parallel to tsetse biology have unraveled the many dependencies partners have for one another. But far less is known mechanistically on how products are swapped between partners and how these metabolic exchanges are regulated, especially to address changing physiological needs. More specifically, how do metabolites contributed by one partner get to the right place at the right time and in the right amounts to the other partner? Epigenetics is the study of molecules and mechanisms that regulate the inheritance, gene activity and expression of traits that are not due to DNA sequence alone. The roles that epigenetics provide as a mechanistic link between host phenotype, metabolism and microbiota (both in composition and activity) is relatively unknown and represents a frontier of exploration. Here, we take a closer look at blood feeding insects with emphasis on the tsetse fly, to specifically propose roles for microRNAs (miRNA) and DNA methylation, in maintaining insect-microbiota functional homeostasis. We provide empirical details to addressing these hypotheses and advancing these studies. Deciphering how microbiota and host activity are harmonized may foster multiple applications toward manipulating host health, including identifying novel targets for innovative vector control strategies to counter insidious pests such as tsetse.

## Tsetse (Diptera: Glossinidae)

**Figure 1:**
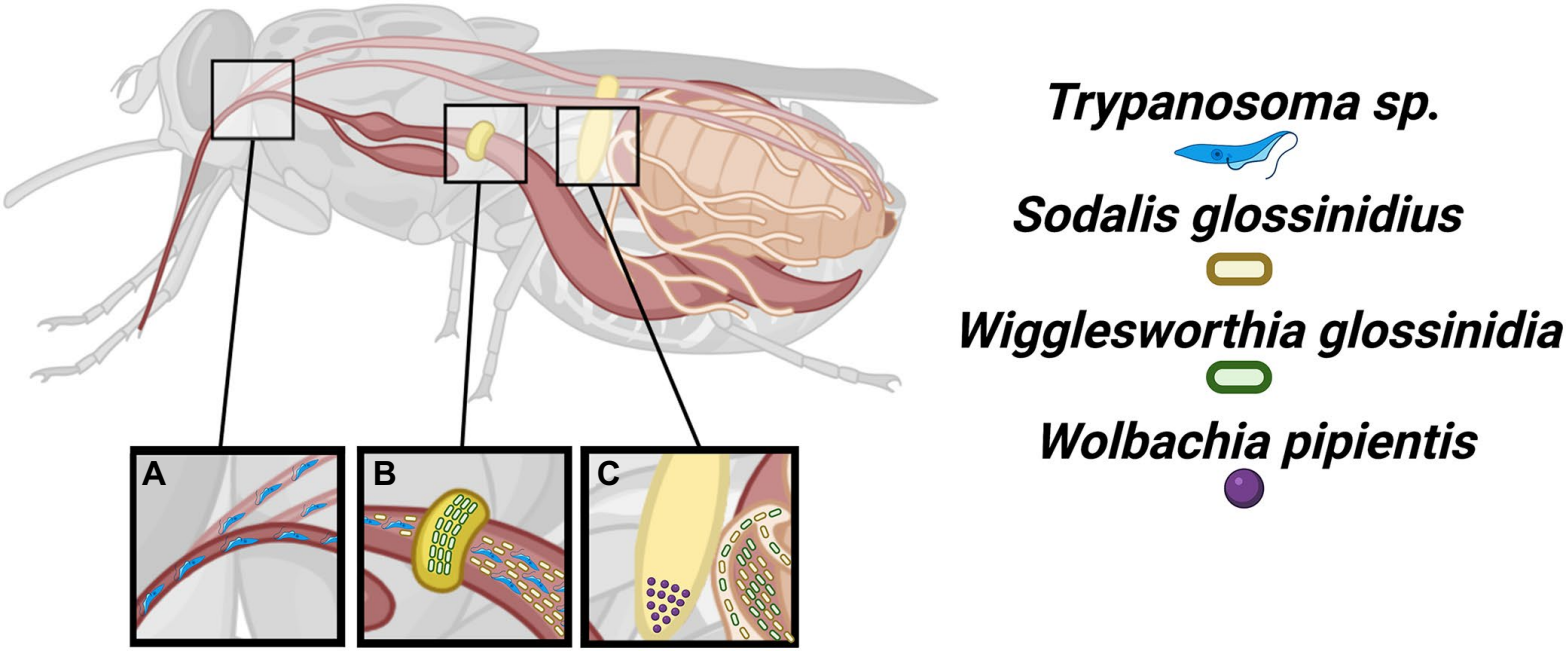
Localization of tsetse microbiota. The tsetse fly is the sole vector of most African trypanosomes. **(A)** These protozoan parasites are introduced into the tsetse fly by an infected blood meal where developmental differentiation, recombination, and migration to the salivary glands occur. **(B)** The *Wigglesworthia* and *Sodalis* symbionts may be found within the bacteriome and gut, respectively. **(C)** The *Wigglesworthia*, *Sodalis*, and *Wolbachia* symbionts are vertically transmitted. The *Wigglesworthia* and *Sodalis* bacteria specifically use milk gland infections while *Wolbachia* infects ovaries for transgenerational persistence.

Tsetse flies are Dipterans belonging to the superfamily of exclusive blood feeders, Hippoboscoidea. Tsetse are exclusively grouped in the family Glossinidae, within the monophyletic genus *Glossina*, and are divided into four groups: morsitans, fusca, palpalis, and austeni (Krafsur, 2009). Tsetse flies are found only in sub-Saharan Africa with the different groups occupying distinct ecological terrains and blood meal preferences which effects the medical and agricultural significance of different species (Solano et al., 2010). Tsetse flies undergo adenotrophic viviparity (Benoit et al., 2015) meaning that a single larva develops *in utero* each gonotrophic cycle (Figure 1). Maternal secretions provide nutrition and seed larva with microbiota (Ma and Denlinger, 1974) through modified female accessory glands known as milk glands.

## Tsetse Fly Microbiota

A key feature in the evolution of eukaryotes has been the spatial and temporal partitioning of biochemical processes for the purpose of regulation (Chomicki et al., 1808; Martin, 2010; Gabaldón and Pittis, 2015). This partitioning reaches even greater complexity with the presence of microbiota and the necessity to coordinate their physiology with host biology particularly if they also rely on vertical transmission for their persistence as this additionally entails coordination with host reproductive biology. Tsetse flies possess a relatively simple core microbiota consisting of three different bacterial species (*Wigglesworthia glossinidia*, *Sodalis glossinidius*, and *Wolbachia pipientis*) varying in their occurrence and ranging in their impact toward host biology from parasitism to mutualism. The obligate mutualist, *Wigglesworthia*, is a focal point of this review and will be further discussed below. A commensal *Sodalis* (Dale and Maudlin, 1999) is not known to impact tsetse fitness but has emerged as a bacterium of interest as a target for paratransgenesis and introducing a trypanosome refractory phenotype (De Vooght et al., 2018). Lastly, *W. pipientis* (supergroup A) may be harbored by tsetse typically within reproductive tissues (O’Neill et al., 1993; Cheng et al., 2000; Balmand et al., 2013) which may result in cytoplasmic incompatibility between mating of differentially infected individuals (Alam et al., 2011).

## Parasitic Trypanosomes

Tsetse flies are the obligate vectors of most African Trypanosomes, *Trypanosoma* species, with an association dating back about 35 million years (Steverding, 2008). Trypanosome parasites are the causative agent of Human African Trypanosomiasis (HAT; *T. brucei rhodesiense* and *T. b. gambiensis*), a debilitating disease caused by the parasitic invasion of the central nervous system which is lethal if left untreated. The disease is endemic to 36 countries in sub-Saharan Africa. Animal African Trypanosomiasis (AAT; *T. b. brucei*, *T. vivax,* and *T. congolense*) is a wasting disease caused by trypanosome infections of domestic animals, contributing to food insecurity within impacted areas. Trypanosome infections of tsetse impose a reproductive burden on females (Hu et al., 2008) likely due to competition for resources with some of these provided by the microbiota (Michalkova et al., 2014; Rio et al., 2019).

## The Obligate Tsetse Mutualist *Wigglesworthia glossinidia*

Both sexes of tsetse feed exclusively on vertebrate blood and consequently have epidemiological significance toward trypanosome transmission. The blood, although rich in amino acids and iron, is particularly poor in B vitamins (Douglas, 2017), which are essential for animals. The provisioning of multiple B vitamins by the obligate mutualist *W. glossinidia* has enabled the restricted feeding ecology of the tsetse fly. The *Wigglesworthia* symbiont is the most predominant member of the tsetse microbiota (Aksoy, 1995; Chen et al., 1999; Aksoy et al., 2014; Tsagmo Ngoune et al., 2019) and inhabits the cytosol of specialized tsetse epithelial cells known as bacteriocytes that collectively form a bacteriome attached to the anterior midgut (Figure 1). *Wigglesworthia* cells are large, filamentous-like (Aksoy, 1995) and lie free in the cytoplasm, unabated from a host-generated membrane and likely also necessitating unique molecular transfer processes with tsetse.

The tsetse-*Wigglesworthia* association dates to around the incipient stages of species diversification of the Glossinidae family (Aksoy et al., 1995; Chen et al., 1999; Symula et al., 2011). This long interdependence has led to a profound impact on the evolutionary genomics of both species. Host adaptation has involved drastic *Wigglesworthia* genome size reduction (Akman et al., 2002; Rio et al., 2012) tailored to tsetse biology coupled with high genetic drift due to smaller population sizes arising from bottlenecks during vertical transfer. Despite its small size, the *Wigglesworthia* genome retains the potential to synthesize multiple B complex vitamins, namely, thiamine (B_1_), riboflavin (B_2_), nicotinamide (B_3_), pantothenic acid (B_5_), pyridoxine (B_6_), and folate (B_9_; Akman et al., 2002; Rio et al., 2012) believed to complement metabolic deficiencies in the blood feeding ecology of tsetse. To date, *Wigglesworthia* has not been cultured but with the availability of annotated genomes (Akman et al., 2002; Rio et al., 2012) and the advent of innovative culture technologies (Lagier et al., 2018; Cross et al., 2019) this may ultimately be achieved opening up a wide array of research questions. Additionally, the availability of an extracellular population of *Wigglesworthia* within maternal milk gland secretions (Ma and Denlinger, 1974; Attardo et al., 2008; Balmand et al., 2013) may also facilitate culturing.

As obligate mutualists, tsetse rely on *Wigglesworthia* for the optimal performance of several physiological processes involved in nutrition, digestion, immunological maturation and reproduction (and likely the connection between these; Wang et al., 2009; Snyder et al., 2010; Weiss et al., 2011, 2013; Michalkova et al., 2014; Snyder and Rio, 2015). In support of its specialization, the bacteriome is enriched in fly gene transcripts that belong to the transmembrane category (Bing et al., 2017; Medina Munoz et al., 2017, 2021), which includes amino acid transporters and multivitamin transporters, likely facilitating nutrient exchange between tsetse and *Wigglesworthia*. In turn, *Wigglesworthia* transcripts are enriched for the metabolism of cofactors and vitamins, supporting a complementary nutrient synthesis role for uptake by host transporters. Structural and functional examination of transporters, and how these may be regulated by epigenetics will help elucidate mechanisms used for interspecies metabolic regulation, likely involving some type of feedback network based on metabolites crucial for homeostasis, although this remains speculative.

## Epigenetics as Coordinators of Symbiosis

Epigenetics controls gene expression and concomitant phenotype independent of gene sequence (Choudhuri, 2011), thereby enabling a relatively rapid adaptation independent of inheritance. Epigenetic mechanisms within insects include small RNA production (Asgari, 2013, 2015; Lucas and Raikhel, 2013; Zhang et al., 2014a; Lucas et al., 2015), histone post-translational modifications (Dickman et al., 2013; Glastad et al., 2015), chromatin remodeling (Rider et al., 2010; Riparbelli et al., 2012), and DNA methylation (Field et al., 2004). Epigenetics may be heritable but may also be erased and reestablished to address specific environmental cues (Waddington, 2012; Tammen et al., 2013; Bind et al., 2014; Deans and Maggert, 2015; Chatterjee et al., 2018; McCaw et al., 2020; Villagra and Frías-Lasserre, 2020). Our focus in this mini review will be specifically on the roles that microRNAs (miRNAs) and DNA methylation may have toward mediating the coordination of microbe-host interactions.

## microRNAs, Paramount Small Regulatory Elements

miRNAs are small (~22 nt) noncoding RNAs with a primary function in sequence-specific post-transcriptional gene regulation (Ibáñez-Ventoso et al., 2008; Fabian et al., 2010). Gene regulation (generally inhibitory) *via* miRNAs is highly conserved across eukaryotes (Bartel, 2018) with sequence conservation of seed regions (i.e., nucleotides present at positions 2–8 from the 5′ end) facilitating identification across often phylogenetically distant animals (Ligoxygakis et al., 2002; Marco et al., 2010). For example, more than 50% of the characterized *Caenorhabditis elegans* miRNAs are encoded in both human and *Drosophila* genomes (Ibáñez-Ventoso et al., 2008; Asgari, 2011). High conservation of miRNAs among Dipterans has also been observed in studies comparing mosquitoes to *Drosophila* (Lai et al., 2003; Li et al., 2009). Despite this conservation, miRNAs sharing high nucleotide identity may exhibit target variation in different species by undergoing “seed-shifting,” where slight changes in the 5′ end of a miRNA alters the seed region (Wheeler et al., 2009; Marco et al., 2010; Berezikov, 2011), consequentially generating a variety of new mRNA targets. Seed-shifting partnered with duplication is the primary evolutionary force for creating new miRNAs (Bartel, 2009; Berezikov, 2011). While miRNAs are known to be a significant source of regulators of endogenous genes (Carthew and Sontheimer, 2009), their potential role in modulating microbial homeostasis and in preventing dysbiosis has been comparatively understudied.

miRNAs are typically encoded within intergenic regions, in non-coding transcripts, or in rare cases within the coding region of genes (Slack, 2006; Asgari, 2011, 2013). To date, most miRNAs are produced through the canonical pathway, though a rare subset (known as non-canonical microRNAs) do not follow this pathway (Bartel, 2004; Abdelfattah et al., 2014). Canonical miRNA generation begins with transcription by RNA polymerase II of long RNA sequences known as polyadenylated primary transcripts (pri-miRNAs) in the nucleus (Asgari, 2011, 2013). The Drosha-Pasha/DGCR8 complex, also known as the Microprocessor complex, processes and cleaves the stem-loops of the pri-miRNA to form the hairpin precursor miRNA (pre-miRNA; Han et al., 2009; Asgari, 2011, 2013). The ~70 nt pre-miRNA is then transported into the cytoplasm by Exportin 5 and its terminal loop cleaved by Dicer and the *loquacious* protein (mammalian TRBP) forming a ~22 nt miRNA:miRNA* duplex (Asgari, 2011, 2013; Nguyen et al., 2015). Similar to *Drosophila melanogaster* (Tomari and Zamore, 2005), tsetse flies also encode two distinct Dicer proteins (Dicer-1 and Dicer-2) with Dicer-1 required for miRNA production (Lee et al., 2004). Argonaute (Ago) proteins then associate with the miRNA duplex using one of the strands as a guide strand forming a RNA-induced silencing complex (RISC; Kawamata and Tomari, 2010; Nguyen et al., 2015). The remaining strand, miRNA*, is known as the passenger miRNA and may play a regulatory role but is typically degraded (Asgari, 2011, 2013). Dicer cleavage also seems to selectively favor an arm of the precursor stem loop, though this preference can vary in different tissues in a context dependent manner (Griffiths-Jones et al., 2011; Chen et al., 2018; Kim et al., 2020). This variation leads to 3′ or 5′ (typical represented as miRNA -3p or -5p) isomiRs being present for miRNAs (Kim et al., 2020). This arm shifting is also responsible for generating a large portion of the diversity within miRNA families (Okamura et al., 2008; de Wit et al., 2009; Berezikov, 2011; Griffiths-Jones et al., 2011).

It is likely that miRNAs have a diversity of functions within the tsetse fly, as within the related *Drosophila* species a range of roles in development, endocrinology, viral immunity, and behavior have been described (Carthew et al., 2017). This is further supported by the conservation of many miRNAs homologs in the more distantly related mosquitos, although miRNA conservation does not necessarily suggest functional retention since some miRNAs are also predicted to have numerous targets (Lai et al., 2003; Friedman et al., 2009; Li et al., 2009). Of relevance, in *Anopheles gambiae* mosquitoes, an elevated abundance of miR-305 is known to increase susceptibility toward *Plasmodium* infections (Dennison et al., 2015), likely mediated by disrupting mRNAs involved in metabolic (Lampe and Levashina, 2018) and immunological processes (Dennison et al., 2015). Similarly, shed trypanosome VSG surface coat antigen when internalized by tsetse cardia cells, decreases miR-275 expression within the midgut (Aksoy et al., 2016; Vigneron et al., 2018). Consequently, the reduction of miR-275 results in compromising the synthesis of the peritrophic matrix (PM) by inhibiting peritrophin expression, the Wnt-signaling pathway and Iroquois/IRX family of transcription factors in the cardia thereby disrupting digestion and strengthening vector competence (Aksoy et al., 2016). As a proof of principle, paratransgenic *S. glossinidius* engineered to express tandem antagomir-275 repeats (3xant-*miR275*) phenocopies the compromised peritrophic matrix and offers an exciting (and economical) technological advancement toward studying the regulatory roles of other miRNAs. Lastly, tsetse with symptomatic Salivary Gland Hypertrophy Virus (SGHV) infections exhibit different tsetse miRNA and SGHV miRNA expression profiles upon comparison to asymptomatic flies. With symptomatic flies, the most highly expressed miRNAs are predicted to target immune-related mRNAs, including those encoded by fibrillin-1 (*FBN1*) and Ras-related protein-27 (*Rab27*), and others involved in reproduction such as apolipoprotein lipid transfer particle (*Apoltp*) and vitellogenin receptor (*Vtgr*; Meki et al., 2018). These genes are all downregulated within symptomatic flies contributing to viral immune evasion and associated ovarian aberrations and loss of reproductive fitness (Abd-Alla et al., 2010).

In previous insect research low or absent miRNA homology suggests novel biological or physiological functions of that miRNA (Marco et al., 2010). Using a custom pipeline of bioinformatic tools on publicly available tsetse Expressed Sequence Tags (ESTs), 10 miRNAs were found to be unique to tsetse flies with gmr-miR 619-5p and gmr-miR-2490-3p predicted to target genes impacted by trypanosome infection, including those encoding the thioester-containing protein (Tep-1) and heat shock protein 60A (Hsp60a; Yang et al., 2020), although experimental validation of molecular regulation remains to be shown.

miRNAs may also directly impact microbiota composition and activity (Ibáñez-Ventoso et al., 2008; Friedman et al., 2009). Besides pathogenic associations, miRNAs are also involved in the regulation of essential members of the microbiota. For example, with the symbiosis between aphids and their symbiont *Buchnera* (a similar ancient obligate nutritional mutualism to the tsetse-*Wigglesworthia* association; Douglas, 1998; Feng et al., 2019), 14 aphid-generated miRNAs are evolutionarily conserved among phylogenetically distant aphid species with significantly different expression of these within bacteriomes relative to symbiont-free tissue (Feng et al., 2018) strongly supporting roles in mediating symbiosis. Moreover, 84 mRNA targets with a predominant function in the principal functional role of the symbiosis, amino acid transport and metabolism (Feng et al., 2018), were identified as putative targets of these miRNAs. At least 10 of the 14 miRNAs have been identified to be of importance toward other host–microbe interaction studies (Skalsky et al., 2010; Jayachandran et al., 2013; Mehrabadi et al., 2013; Mayoral et al., 2014a; Zhang et al., 2014b; Jin et al., 2017; Qiang et al., 2017; Liu et al., 2019) suggesting a universal (and likely convergent) role in the regulation of symbioses. Compellingly, research in tsetse has indicated genes associated with both amino acid transport and metabolism (*Wigglesworthia* is auxotrophic for the majority of amino acids) have differential expression in aposymbiotic compared to wild-type flies, which may indicate a similar regulatory role toward these genes could be played by tsetse miRNAs (Medina Munoz et al., 2017).

A plethora of questions remain about whether animals can use miRNAs to impact gene expression in microbes. Previous research on miRNAs in insect microbial relations has focused on identifying miRNAs produced by the host and assumed to target host mRNAs involved in the symbiosis (Carthew et al., 2017; Feng et al., 2018, 2019). Encouraging research that suggests targeting of microbial (particularly bacteria) RNA may in fact be plausible comes from studies demonstrating miRNAs regulating mitochondrial mRNAs (Li et al., 2012; Duarte et al., 2014; Macgregor-Das and Das, 2018). Mitochondria, as remnants of an ancient Alphaproteobacterium endosymbiont rendered modern-day organelle, still retain a double membrane (Macgregor-Das and Das, 2018) similar to *Wigglesworthia* (Aksoy, 1995). If tsetse miRNAs interact with *Wigglesworthia* to coordinate gene expression, they are likely not alone. It is possible that other mutualists with significantly reduced genomes such as *Wigglesworthia* may also rely on these small RNAs as opposed to proteins for gene regulation, representing a novel avenue for experimental exploration to further our understanding of intracellular signaling (Hansen and Degnan, 2014). Lastly, a further compelling and reciprocal research focus is whether small RNAs encoded by bacterial mutualists may manipulate host genes, which is not unknown of within Gammaproteobacteria. For example, intracellular *Salmonella* produce a miRNA-like Sal-1 processed by human AGO2 proteins which enhances intracellular *Salmonella* survival (Gu et al., 2017). Further the production of a *Wolbachia* small noncoding RNA, *W*snRNA-46A, enhances the transcription of *Aedes aegypti* Dynein heavy chain (*Dhc*) which facilitates *Wolbachia* association with microtubules enabling its transfer during mosquito oocyte or embryonic development (Mayoral et al., 2014b). Whether small noncoding RNAs produced by *Wigglesworthia* may impact tsetse metabolism or immunity remains to be seen.

## DNA Methylation as a Regulatory Conduit Between Microbiota and Host Physiology

DNA methylation is the addition of methyl (CH_3_) groups to cytosine residues (5mC) typically within 5′-cytosine-phosphate-guanine-3′ (CpG) dinucleotides (Lyko, 2018). Across insect taxa, genome methylation exhibits a patchy distribution and differs relative to those of vertebrates in regards to general localization (Head, 2014). For example, DNA methylation is prevalent in the promoter regions (creating CpG islands) of vertebrate genomes, with modifications altering the interactions of transcription factors and histones *via* steric effects (Moore et al., 2013). Within insect genomes, DNA methylation is pervasive within gene bodies (Cingolani et al., 2013; Takayama et al., 2014; Jeong et al., 2018; Huang et al., 2019), where it is involved in alternative gene splicing and the creation of isoforms (Lyko et al., 2010; Bonasio et al., 2012; Terrapon et al., 2014). Although CpG methylation is also present within insect genomes, methylation is more prevalent in the CpA and CpT dinucleotide contexts (Takayama et al., 2014). For example, splice junctions are enriched for non-CpG methylation (Cingolani et al., 2013) in bees and different splice variants of the same gene are associated with diverse methylation patterns (Lyko et al., 2010).

DNA methylation is among the most amenable epigenetic modifications to identify given its relative ease in identification. For example, commercially available antibodies detect the presence of methylated nucleotides within genomic DNA (Kunert et al., 2003) and may be used to enrich for methylated DNA prior to high-throughput sequencing (Glastad et al., 2014). Moreover, bisulfite sequencing and subsequent mapping (Ku et al., 2011), enables the characterization of nucleotide methylation across a reference genome of interest, permitting the discovery of preferential motifs (Takayama et al., 2014; Panikar et al., 2015). The generation of reference DNA methylomes for a variety of insects through developmental stages with validated ties to phenotypes will greatly facilitate our understanding of epigenetic modifications toward insect biology and fuel future research endeavors.

## The Role of Folate Toward DNA Methylation

Although vitamins are essential to physiology, animals lack the ability to synthesize these *de novo* and must either obtain these critical nutrients through diet and/or microbiota provisioning (Brecher and Wigglesworth, 1944; Douglas, 2017). Folate (B_9_) is particularly deficient within blood (Brecher and Wigglesworth, 1944; Edwards et al., 1957; Pietrzik et al., 2010; Nikoh et al., 2014; Douglas, 2017; Duron et al., 2018), with symbiotic bacteria often provisioning this essential cofactor to strictly hematophagous animals (Duron and Gottlieb, 2020). A significant role for *Wigglesworthia* within their hosts is the production and provisioning of folate, which is critical for tsetse reproduction and larval development while also serving to enhance vector competence (Snyder and Rio, 2015; Rio et al., 2019).

Folate is necessary for DNA methylation because it is transformed into 5-methyltetrahydrofolate (5-methylTHF), needed for the formation of methionine from homocysteine (Crider et al., 2012). Once methionine has been synthesized, it is joined to ATP and converted into the universal methylation donor *S-*adenosyl methionine (SAM). SAM donates the methyl group during DNA methylation *via* the action of DNA methyltransferases (Crider et al., 2012; Shorter et al., 2015). Folate provisioning by *Wigglesworthia* may provide a means for connecting *Wigglesworthia* metabolism to tsetse genetic regulation *via* DNA methylation. In support of this connection, SAM abundance is significantly decreased in tsetse fly bacteriomes which have been cleared of their *Wigglesworthia* symbionts (Bing et al., 2017).

Due to the lack of DNMT-1 and -3 in the genome, tsetse has been predicted to lack DNA methylation (Bewick et al., 2017). However, due to its close evolutionary relation to *D. melanogaster* and the characterization of DNA methylation in the fruit fly genome (Takayama et al., 2014; Panikar et al., 2015; despite also lacking these DNMTs), we hypothesize the presence of methylation in the tsetse genomic DNA, particularly within *Wigglesworthia* harboring bacteriomes which may impact symbiosis activities. Symbiosis altering DNA methylation is not unprecedented in eukaryotes as previously reported in a wide array of organisms including plants (Vannier et al., 2015), anemones (Li et al., 2018), and mice (Warner et al., 1989; Yu et al., 2015), with concomitant changes in symbiosis phenotypes.

## Discussion

**Figure 2:**
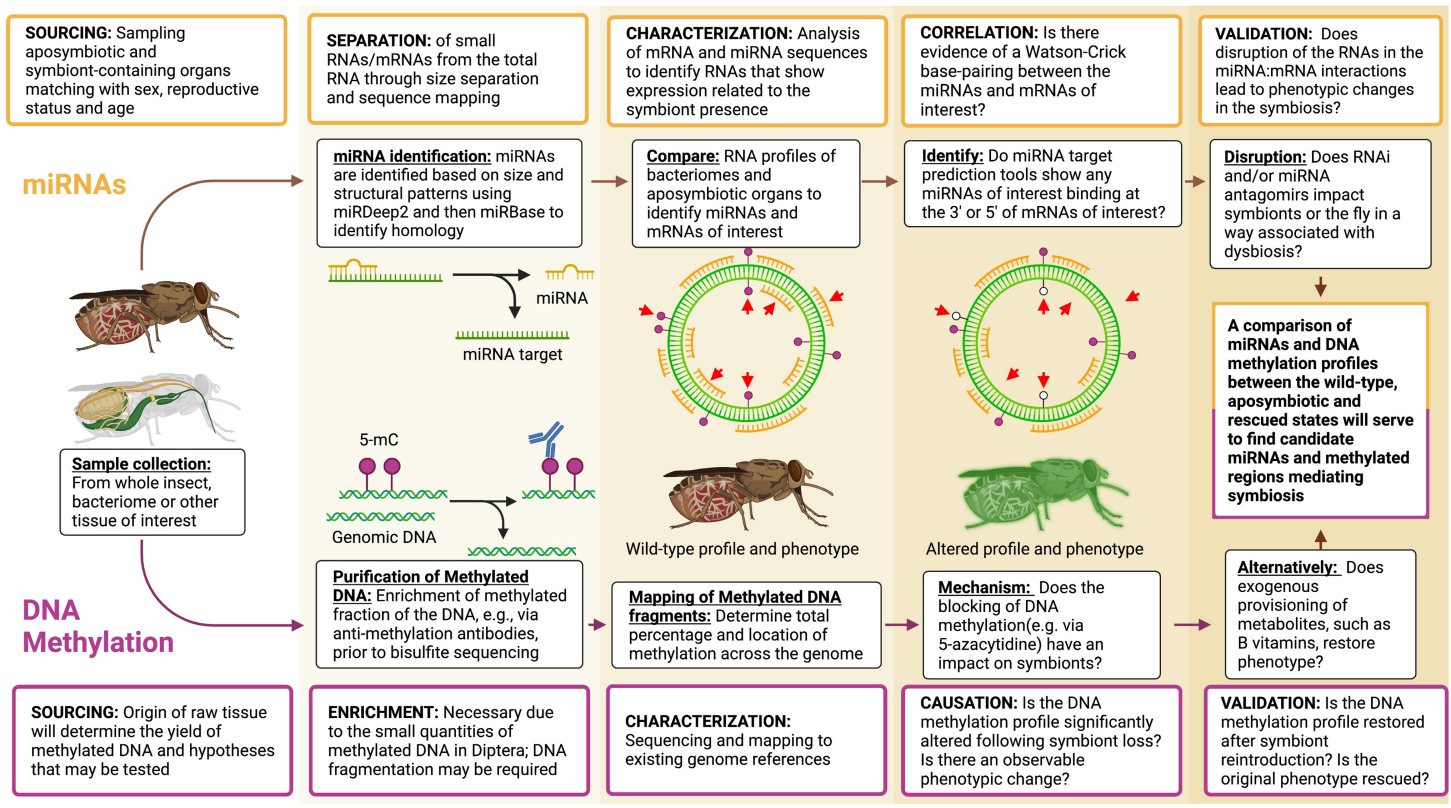
Flow chart for the empirical analyses of epigenetic mechanisms mediating the *Wigglesworthia*-tsetse fly symbiosis. Of primary importance is the establishment of wild-type miRNA and methylation profiles and phenotypes followed by the characterization of altered states. If altered states are fully or partially rescued by restoration of the epigenetic mechanism through either the reintroduction of the symbionts and/or their provisioned metabolites, then there is evidence in favor of epigenetics mediating symbiosis. Distinct branches highlight methodology for the detection of miRNAs and DNA methylation impact toward symbiosis, while smaller boxes feature comments on the relevant methodology. Red arrows indicate differences in nucleic acid profiles upon comparison of sample groups.

Metabolite provisioning is a fundamental role of host-associated microbiota, particularly of animals with limited diets such as the strictly blood feeding tsetse fly. The tsetse fly provides a valuable, and medically significant, model system to dissect regulatory mechanisms that coordinate host-microbiota activities, including nutrient exchange, immunological maturation and vector competence. Much has been gathered on the composition, functional contribution and evolutionary history of the tsetse microbiota, yet little is known regarding mechanisms coordinating microbial activity with host biology. Here we emphasize the investigation of epigenetics, specifically the role of miRNAs and DNA methylation, toward regulating interspecies activities as these may deliver rapid cues for the restoration and maintenance of homeostasis through tsetse development and following perturbations. We provide support for further investigations of these regulatory mechanisms and experimental guidance (Figure 2) for the simultaneous characterization of these epigenetic processes and assessing their impact toward the host-microbiota association. Besides providing the basis for a deeper understanding of ecological and organismal biology features and their evolution, the study of symbioses and its regulation, particularly in blood-feeding vectors is of significant consequence for epidemiological studies and the design of control strategies aimed at halting transmission of vector-borne diseases.

## Author Contributions

All authors listed have made a substantial, direct, and intellectual contribution to the work and approved it for publication.

## Funding

We acknowledge the support for writing this review by a WVU Eberly College Faculty Development Grant. The tsetse miRNA work done in our laboratory is supported by NIH-NIAID R21AI145271 (RR). Figures were created through BioRender.

## Conflict of Interest

The authors declare that the research was conducted in the absence of any commercial or financial relationships that could be construed as a potential conflict of interest.

## Publisher’s Note

All claims expressed in this article are solely those of the authors and do not necessarily represent those of their affiliated organizations, or those of the publisher, the editors and the reviewers. Any product that may be evaluated in this article, or claim that may be made by its manufacturer, is not guaranteed or endorsed by the publisher.

## Appendix

### Current research gaps

1. *Wigglesworthia*’s tight integration in tsetse physiology requires mechanisms for the regulatory control of population size and function. Intriguingly, *Wigglesworthia* lies free in the cytoplasm of bacteriocytes which may facilitate tsetse miRNAs to interact with these symbionts. Are tsetse miRNAs localized to the bacteriome acting on *Wigglesworthia* transcripts to control expression? Due to differences in nutrient demands during pregnancy and through aging, how may tsetse miRNA expression be impacted particularly toward the metabolic integration of *Wigglesworthia* symbionts?
2. *Salmonella* produce small RNAs which alter the host phenotype and lead to increased virulence. The DNA sequence which produces these small RNAs may also be found in many other Gamma-proteobacteria, making it plausible that these microRNA-like small RNAs have homologous roles in other bacteria. Do *Wigglesworthia* produce miRNA-like small RNAs to create favorable environments in the fly? Small bacterial RNAs represent an additional avenue for exploration toward advancing our understanding of interkingdom communication.
3. What types of epigenetic mechanisms may regulate the influx/efflux of substrates at symbiont and host transporters which lie at the interface of the association?
4. Pathogenic bacterial infections are associated with changes in the DNA methylation of several insects including members of Diptera (Ye et al., 2013; LePage et al., 2014), Lepidoptera (Baradaran et al., 2019), and Hemiptera (Negri et al., 2009). May beneficial symbionts also impact host DNA methylation? Establishing a cause-effect relationship and biochemical steps involved in these outcomes will be essential toward our understanding of the regulatory role exerted by bacteria on insect physiology.
5. What other epigenetic mechanisms may be affected by the metabolites of microbiota? For example, a role in epigenetics through biotin (B7) provisioning by symbionts may also occur given that histone biotinylation plays a role in transcriptional repression of genes and DNA repair.
